# Chronic Unpredictable Stress (CUS)-Induced Anxiety and Related Mood Disorders in a Zebrafish Model: Altered Brain Proteome Profile Implicates Mitochondrial Dysfunction

**DOI:** 10.1371/journal.pone.0063302

**Published:** 2013-05-14

**Authors:** Sumana Chakravarty, Bommana R. Reddy, Sreesha R. Sudhakar, Sandeep Saxena, Tapatee Das, Vuppalapaty Meghah, Cherukuvada V. Brahmendra Swamy, Arvind Kumar, Mohammed M. Idris

**Affiliations:** 1 Chemical Biology Division, CSIR-Indian Institute of Chemical Technology (IICT), Hyderabad, India; 2 CSIR-Centre for Cellular and Molecular Biology (CCMB), Habsiguda, Hyderabad, India; Tulane University Medical School, United States of America

## Abstract

Anxiety and depression are major chronic mood disorders, and the etiopathology for each appears to be repeated exposure to diverse unpredictable stress factors. Most of the studies on anxiety and related mood disorders are performed in rodents, and a good model is chronic unpredictable stress (CUS). In this study, we have attempted to understand the molecular basis of the neuroglial and behavioral changes underlying CUS-induced mood disorders in the simplest vertebrate model, the zebrafish, *Danio rerio*. Zebrafish were subjected to a CUS paradigm in which two different stressors were used daily for 15 days, and thorough behavioral analyses were performed to assess anxiety and related mood disorder phenotypes using the novel tank test, shoal cohesion and scototaxis. Fifteen days of exposure to chronic stressors appears to induce an anxiety and related mood disorder phenotype. Decreased neurogenesis, another hallmark of anxiety and related disorders in rodents, was also observed in this zebrafish model. The common molecular markers of rodent anxiety and related disorders, corticotropin-releasing factor (CRF), calcineurin (ppp3r1a) and phospho cyclic AMP response element binding protein (pCREB), were also replicated in the fish model. Finally, using 2DE FTMS/ITMSMS proteomics analyses, 18 proteins were found to be deregulated in zebrafish anxiety and related disorders. The most affected process was mitochondrial function, 4 of the 18 differentially regulated proteins were mitochondrial proteins: PHB2, SLC25A5, VDAC3 and IDH2, as reported in rodent and clinical samples. Thus, the zebrafish CUS model and proteomics can facilitate not only uncovering new molecular targets of anxiety and related mood disorders but also the routine screening of compounds for drug development.

## Introduction

Stress, which is becoming more prevalent in daily human life, has dual impacts on brain and body physiology. Within limits, it has positive effects, such as improved memory performance [1] and increased alertness, focus, and energy; furthermore, it can help people to cope with unfavorable situations. However, chronic stress takes a toll on physiology, as well as on mood, affecting productivity and quality of life and ultimately leading to affective disorders, such as anxiety, depression and other related disorders, which are termed mood disorders [2]–[5]. During the last few decades, researchers using animal models have found that chronic stress can induce depression, anxiety and related mood disorders [2], [3], [6]. Chronic unpredictable stress (CUS), one of the most clinically relevant stress paradigms in rodents, mimics a number of behavioral characteristics observed in patients with anxiety, depression and related mood disorders [6], [7]. Knowing that the initial response to chronic stress is anxiety followed by depression and that the co-morbidity of these mental disorders is an undeniable risk factor for other bodily diseases, such as inflammatory bowel disease [6], [8], [9], it is imperative that we develop simpler models to study the effects of chronic stress on the brain and to identify associated molecular markers that can be effectively used for CNS drug screening.

Most of the recent experimental studies of rodent models appear be costly affairs as far as screening of thousands of novel, potential candidate small molecules for CNS drug discovery is concerned. In recent years, zebrafish have attracted interest as a useful tool for the *in vivo* screening of compound libraries for various diseases due to their ease of handling, lower cost of maintenance, and faster reproduction [10]–[12]. Extensive research efforts are ongoing in neurobehavioral research using this animal model for several reasons, most importantly, their high genetic homology with humans [10], [11]. A few research groups [13]–[15] have begun to use zebrafish as a model for anxiety and related mood disorders. However, only a few behavioral assays for mood disorder phenotypes have been established, and in zebrafish brain, the molecular changes underlying mood disorders have not been characterized. In the present study, we aimed to establish a robust stress-induced zebrafish model for anxiety and related disorders, mimicking affective disorders in rodents and humans, and to use proteomics to uncover relevant molecular markers.

## Materials and Methods

### Animals and Housing

Adult zebrafish were bred and raised in captivity. All of the animals were raised in large tanks with a natural day light/dark cycle and two feedings until they arrived in the laboratory. In the fish laboratory, the animals were acclimatized to the experimental room conditions by maintaining them at 28±2°C, 14/10 h light/dark cycle, three feedings and constant aeration [16]. A total of 120 male and female adult zebrafish in the 6–12 month age group were selected for the study. After the habituation period, the animals were grouped as control and test sets (n = 30 per group) in duplicates, and the test animals were subjected to two different stressors per day for a period of 15 days. All animal procedures were approved by the Institutional Animal Ethics Committee (IAEC/CCMB/Protocol No.11/2012).

### Chronic Unpredictable Stress (CUS)

The animals were subjected to a variety of chronic stressors, such as restraint stress (RS), social isolation (SI), over-crowding (OC), tank change (TC), cold stress (CS), chasing (C), heat stress (HS), dorsal body exposure (DBE), predator stress (PS) and alarm pheromone stress (APS); the animals were exposed to two stressors daily for 15 days (Table 1). The stressors were administered as follows: each animal was restrained (RS) for an hour in a 2-ml microcentrifuge tube with perforations at both ends for free water flow [12], [15]; exposed to heat stress (HS) and cold stress (CS) by transfer to new tanks maintained at 33°C and 23°C, respectively, for 30 min; socially isolated (SI) in separate beakers for 60 min; over-crowded (OC) with 10 animals in a 250-ml beaker containing only 150 ml water for 60 min; exposed to the animated image of predators (PS) such as *Amatitlania nigrofasciata* and *Monocirrhus polyacanthus* for 60 min in close proximity; kept in housing tanks with low water levels to expose the animal’s dorsal body (DBE) for 2 min; transferred from one tank to another (TC) six consecutive times; and chased (C) by a net for 8 min. For the alarm pheromone stress (APS) administration, the test fish were exposed to water that contained the washings of epidermal cells from euthanized/sliced zebrafish: sliced skin washed for 5 min in a petri dish filled with 10 ml of distilled water, placed on ice and then acutely administered to fresh water in a novel tank into which the study fish were introduced. The fish were subjected to prolonged exposure for a period of 30 min.

**Table 1:** Chronic unpredictable stress paradigm in adult Zebrafish.

		Day1	Day2	Day3	Day4	Day5	Day6	Day7
Week 1	Morning	RS	SI	OC	TC	CS	TC	C
	Evening	HS	CS	DBE	PS	OC	HS	SI
Week 2	Morning	TC	C	DBE	OC	TC	DBE	TC
	Evening	RS	APS	PS	TC	SI	CS	C
Week 3	Morning	APS	NTT/Scototaxis	Collection of brain tissues				
	Evening	DBE						

RS – Restraint Stress; SI – Social Isolation; OC – Overcrowding; TC – Tank change; CS – Cold stress; C – Chasing; HS – Heat stress; DBE – Dorsal body exposure; PS – Predator stress; APS – Alarm pheromone stress; NTT – Novel tank test.

To avoid habituation to stressors, unpredictability was maintained by changing the time and sequence of stressors daily during the 15 days of the stress paradigm. Aeration and temperature were controlled during the presentation of each stressor, except during heating and cooling stresses. The non-stressed control group was maintained in the same room during the 15-day stress period.

### Behavior Testing and Analysis

Twenty-four hours after the CUS paradigm, the novel tank test, shoaling behavior and scototaxis were performed to analyze the behaviors of the control and stressed groups simultaneously. The novel tank test (NTT) mimics the open field and elevated plus maze tests used to assess the anxiety in rodents [17]. After an acclimatization period of 5 min, zebrafish were placed individually in a narrow 15×12×25-cm tank with a water depth of 18 cm divided into three equal, virtually horizontal sections and demarcated by a line on the outside of the tank wall. In the 2-min novel tank test, the time spent by the fish in the different levels of the tank (bottom, middle or upper level) was measured to assess the level of anxiety [17]. A preference for the bottom two levels and less frequent venturing into the upper level of the tank is suggestive of increased anxiety [17]. Similarly, longer latency to enter the upper level, greater numbers of freezing bouts and longer durations in freezing mode indicate the anxious phenotype, as does increased locomotion (number of crosses in the swim area) [15].

In shoal cohesion (SC), an acclimatization period of 10 min was followed by the introduction of zebrafish of both the groups into tanks (three per tank; 15×12×25 cm with 18-cm water level) for shoal response measurement. Shoal cohesion in zebrafish, which is an innate and natural behavior [19] that develops with age [20], also occurs in response to alarming and stressful situations [21]. Recent investigations [12], [15], [21] have suggested a correlation between shoal cohesion and anxiety response.

Scototaxis can be used to assess anxiety in zebrafish and is similar to the dark/light test used to assess anxiety in rodent models [22], [23]. Each zebrafish was placed in water in a central, colorless chamber (dimensions of 4 cm) with sliding doors. As the doors open, the fish can choose to enter the black (dark) or white (light) chamber of the box (40×10×15 cm, *l*×*w*×*h*) with a water level of up to 10 cm. The preference of each fish for the dark and light chambers was recorded over the 5-min test period. Spending longer periods of time in the dark chamber reflects increased anxiety. The anxiety level is also indicated by a decrease in the latency to enter the dark chamber, similar to the light/dark box test for rodents [23].

### Tissue Collection, Protein Preparation and Differential Proteomic Analysis

Zebrafish were anesthetized in 0.004% MS-222 (Tricaine, Sigma-Aldrich) until the gill movement slowed, and then, individual brains were dissected using micro-dissecting tweezers and syringe needles. The extracted brain tissues were washed twice in phosphate-buffered saline, pooled and snap-frozen in liquid nitrogen. Total brain proteins were extracted following homogenization, precipitation and solubilization [24]. Protein was quantified based on the Amido black assay method against BSA [25]. Differential proteomic analyses between the control and test (CUS) samples were performed using 2-dimensional gel electrophoresis (2DE) and mass spectrometric analysis. The first dimension of separation, which was based on pH, was performed over a broad range using 3–10 NL IPG strips and 300 µg of total proteins; these analyses were performed in triplicate. The second dimension of separation, which was based on mass, was accomplished using 12% SDS-PAGE. The gels were stained with colloidal Coomassie and then scanned. The 2DEs of the control and the test samples were analyzed for differential analysis using the IMP software (GE Healthcare). Stained gel spots exhibiting an increase or decrease exceeding 1.5-fold between the control and test samples were eluted, processed and trypsin-digested for MS and MS/MS analyses based on Fourier transform mass spectrometry (FTMS) and ion trap tandem mass spectrometry (ITMSMS) using the Orbitrap Nano analyzer (Thermo) [26]. The proteins were identified from the obtained MS/MS peak list by searching against the *Danio rerio* database (FASTA v.3.71); the identifications were confirmed using the experimental mass and pI data.

### SDS-PAGE & Immunoblotting

Immunoblotting was performed to validate a subset of the differentially regulated proteins, including voltage-dependent anion channel 3 (VDAC3) and beta-synuclein (SNCB). Additionally, immunoblotting was performed for pCREB to determine whether this molecular marker of the rodent mood disorder model is also relevant in the zebrafish model. Control and stressed brain protein samples were individually denatured and separated on a 10% polyacrylamide gel (40 µg per lane). The separated proteins were then transferred to PVDF membranes at 100 mA and 4°C. The blot was blocked with fresh blocking buffer (5% nonfat milk and 0.1% Tween-20 in PBS, pH 7.4) for 1 hour followed by incubation with the appropriate primary antibody (Millipore, 1∶1000 dilution) overnight at 4°C. After washing the blot with PBS (containing 1% Tween), each membrane was incubated with the appropriate horseradish peroxidase-conjugated secondary antibody (1∶10000) at room temperature. Finally, after PBS washes, the bands were visualized with a Gel Doc enhanced system. The band intensities were analyzed with ImageJ software.

### Gene Expression Analysis by Real-time PCR

Total RNA was extracted from the pooled adult zebrafish brains using TRI reagent (Sigma). cDNA was synthesized from 1 µg of total RNA by reverse transcription (SuperScript III -Invitrogen) using random hexamers. Quantitative real-time PCR analyses were performed for the genes that code for CRF, calcineurin and BDNF, which are molecular markers that are commonly observed in rodent models of anxiety and related mood disorders. PCR was also performed for the genes that code for Prohibitin2 and SLC25A5, two of the 18 proteins found to be maximally regulated (at least 2.5-fold) in our proteomic data. These proteins, which are involved in mitochondrial function, are also implicated in neuropsychiatric and neurodegenerative disorders [27]. All qPCR reactions were performed in an ABI 7900HT (Applied Biosystems) using MESA green Q-PCR master mix plus for SYBR assay (Eurogentec). PCR was performed in triplicate using gene-specific primers (shown in Table 2), which were designed and synthesized per the requirements of RT-qPCR. The relative abundance of mRNA was determined by normalization to β-actin levels, and the results were expressed as relative expression levels. The data were quantified by the 2^−ΔΔCt^ method.

**Table 2:** List of primers used in quantitative real-time PCR amplification.

Gene Name	Accession Number	Primer Sequence	
		Forward (5′–3′)	Reverse (5′–3′)
CRF	BC164878	CATCCCAGTATCCAAAAAGAGC	TCGTAGCAGATGAAAGGTCAGA
Calcineurin (Ppp3r1a)	XM687678	GCCTTTAGGATCTACGACATGG	ATATTCTCCCGTCTCCGTCTTT
BDNF	NM131595	GGACAAAAAGACGGCAATAGAC	CGATCTTCCTTTTGCTATCCAT
PHB2	NM199681	AGAAGCCACATATACAGTGGAG	AGACAACACACGTAGACCAATG
SLC25A5	BC152234	CATCATTTACAGAGCTGCCTAC	TTTACGTCCAGACTGCATCATC
VDAC 3	BC153608	ATGAACGTGGGCTGTGATCTAG	CCGAACTCTGTGCCATCATTAAC
β - Actin	AF025305	GGCATCACACCTTCTACAATGA	TACGACCAGAAGCGTACAGAGA

### BrdU Immunostaining

BrdU incorporation was performed according to Yeo and Chitnis [28]. The adult zebrafish were kept in 10 mM BrdU (5′-bromo-2′deoxyuridine, Sigma) solution for 4 hours to allow for the uptake of thymidine analog by the zebrafish, presumably via the gills. The fish were returned to normal water that was changed twice at 2 h intervals to minimize the reuptake of excreted BrdU. Coronal sections (35 µm thick) were obtained from the telencephalon, optic tectum, and other brain areas (Leica cryostat # CM3050). The sections were processed for immunostaining (SuperPicture IHC detection kit, Invitrogen) per the manufacturer’s protocol. Briefly, the sections were pre-treated with 50% formamide in 2X SSC to denature the DNA. After antigen retrieval and peroxide quenching, the sections were blocked in 10% horse serum overnight in primary antibody (anti-Mouse BrdU, Calbiochem, 1∶500), followed by brief incubation in secondary antibody and diaminobenzidine (DAB) treatment. Then, the sections were washed, dehydrated in a graded alcohol series and mounted, and the stain intensity was visualized under microscope. The images of the bromodeoxyuridine (BrdU)-reactive cells were obtained at 20X magnification using a microscope (Carl Zeiss, Germany).

### Statistical Analysis and Figure preparation

Data are expressed as the means ± SEM. Differences between the control and stressed groups were evaluated using the two-tailed unpaired Student’s t test. Figures and images were generated and edited using Adobe Photoshop 7.0.

## Results

### Repeated CUS-induced Anxiety and Related Mood Disorder Phenotype in Zebrafish

**Figure 1:**
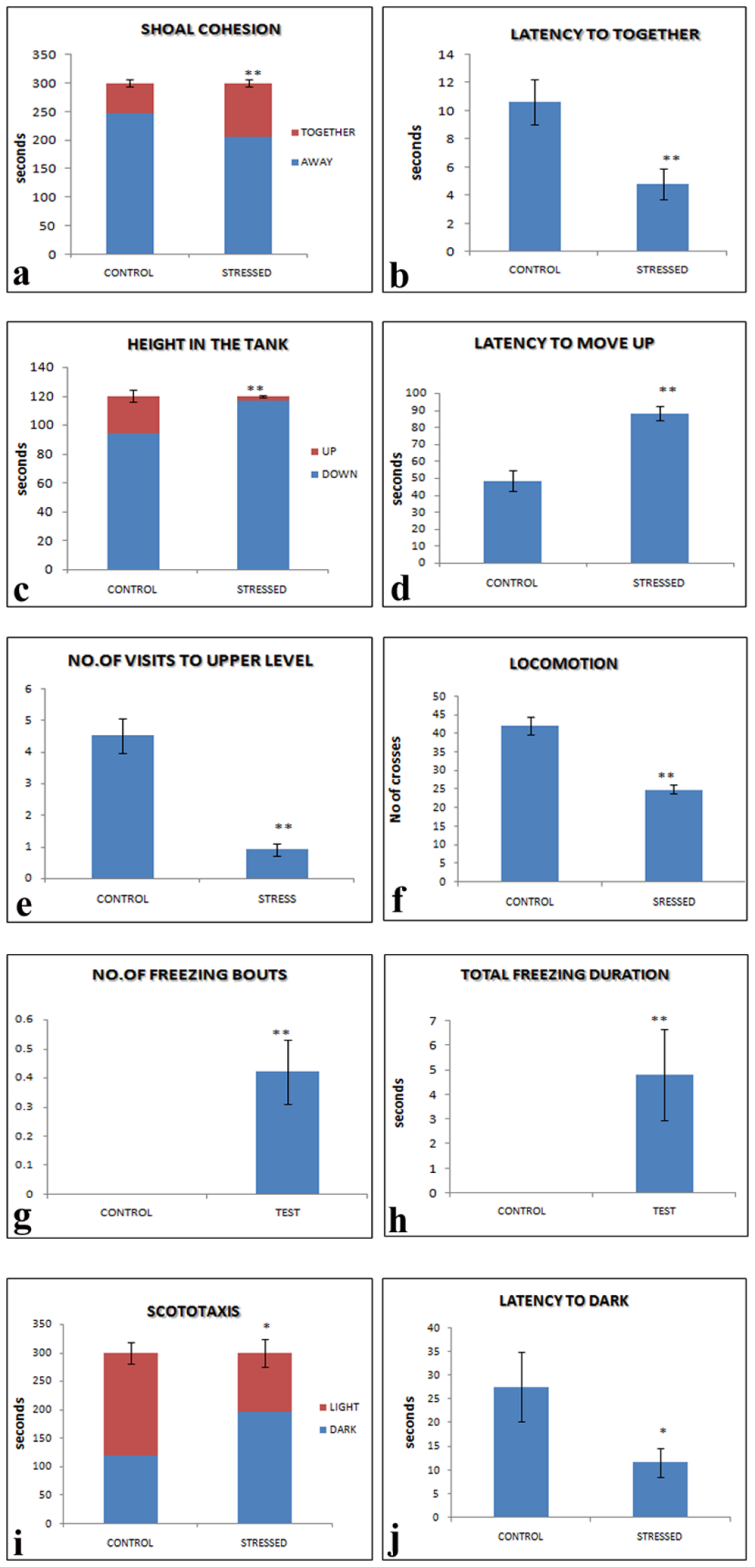
CUS induces anxiety and related mood disorder phenotype in zebrafish. Effects of 15 days of chronic unpredictable stress (CUS) on shoal cohesion (a & b), preferred swim level (c & d), locomotion (e & f), freezing bouts (g & h) and scototaxis (i & j) parameters in zebrafish. Data are expressed as the mean ± SEM. *p<0.05 vs. control group, **p<0.01 vs. control group, (independent *t*-test). n = 30 per group.

Exposure to CUS for 15 days induced alterations in fish behavior that reflected an anxiety and related mood disorder phenotype, as shown in Figure 1 (a–j). Chronically stressed fish showed significantly altered shoal cohesion (Figure 1a & b). The total duration of shoal cohesion increased significantly in the CUS group compared to non-stressed control group. There was also a significant reduction in the latency to shoal cohesion among the chronically stressed test fish compared to the control non-stressed fish, suggesting CUS-induced hyper-anxiety. In the novel tank test, stressed fish spent significantly more time in the bottom two levels of the tank (p<0.01) than in the upper level of the tank. There was also a significant increase in the latency to enter the upper level of the tank (p<0.01) in the CUS group compared to that in the control group. In addition, all of the fish in the CUS group showed frequent freezing bouts upon reaching the upper level of the tank, and the total duration of the freezing mode was fairly high, whereas in the control group, the freezing response was absent. Thus, the NTT behavioral outcome also reflected a hyper-anxiety phenotype in response to CUS. The locomotion of stressed fish was significantly lower than that of the control group, as shown by the significant decrease in the number of crosses in the swim area (Figure 1e & f); this decrease appeared to be due to frequent freezing bouts upon entering in the upper part of the tank (Figure 1g & h). In the scototaxis test, the total time spent in the dark chamber was significantly higher in stressed animals than in the non-stressed controls (Figure 1i & 1j). In addition, the latency to enter the dark chamber also decreased significantly in stressed fish compared to non-stressed ones. Thus, the results of the shoal cohesion, novel tank and scototaxis tests suggest that the zebrafish developed an anxiety and related mood disorder phenotype following 15 days of chronic unpredictable stress [Table 3].

**Table 3:** Summary of behavioral analyses.

Behavior analyzed	Stress vs. Control	
Shoal cohesion	↑	
Latency to shoal cohesion	↓	
Preferred swim zone	Upper tank level ↓	Bottom tank level ↑
Latency to move up	↑	
Number of visits to upper levels	↓	
Locomotion	↓	
Number of freezing bouts	↑	
Freezing duration	↑	
Scototaxis	Light ↓	Dark ↑
Latency to dark	↓	

### CRF, Calcineurin, and pCREB are Molecular Markers for Anxiety and Related Mood Disorders in Zebrafish, Like Rodent Models

**Figure 2:**
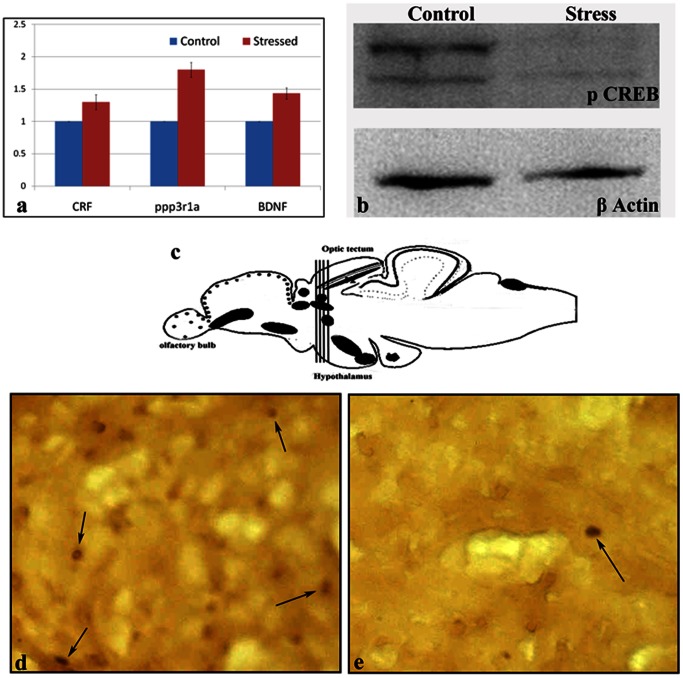
CUS in zebrafish mimics established molecular markers of rodent mood disorder models and attenuates adult neurogenesis. Effects of the 15-day chronic unpredictable stress (CUS) protocol on the levels of CRF, ppp3r1a (calcineurin) and BDNF, which are known molecular markers in rodent mood disorder models. There is significant upregulation in the transcript level of these genes in stressed group, compared to control (a & b). Neurogenesis in the optic tectum (highly proliferating neurogenic niche) of the zebrafish brain (c, d & e) is adversely affected after CUS, as BrdU positive cells are rarely observed in the stressed sample.

After establishing that 15 days of repeated stress could successfully induce an anxiety disorder phenotype, we evaluated zebrafish brains for a few of the most common molecular markers of anxiety and related disorders reported in rodent models, such as corticotrophin-releasing factor (CRF), calcineurin and phospho CREB (cyclic AMP response element binding protein) [3], [29] . Interestingly, zebrafish brain had significantly higher mRNA expression levels for *CRF* and *calcineurin* compared to the levels in unstressed control brain (Figure 2a) and reduced pCREB (Figure 2b), as has been shown in rodent models. Surprisingly, another established molecular marker that is downregulated in most of the brain regions of depressed rodents, brain-derived neurotrophic factor (BDNF) [2], [30], did not show significant downregulation in zebrafish brain but was instead significantly up-regulated. This finding may have been the result of using whole fish brain for gene expression analysis rather than specific brain areas, as in rodent models.

### The Reduced Adult Neurogenesis Observed in Rodent Stress-induced Anxiety Disorder Models is Replicable in Zebrafish

Reduced adult hippocampal neurogenesis in the dentate gyrus (DG) is a very striking feature that has been reported in rodent models of stress-induced anxiety, depression and related mood disorders [31], [32]. We therefore investigated the effects of CUS on adult neurogenesis in the corresponding brain area in fish, the telencephalon [33], and another highly neurogenic niche in the fish brain, the optic tectum. CUS substantially reduced the rate of proliferation in the neurogenic progenitor population in the optic tectum (Figure 2d & e) and the telencephalon (Figure S1a, b), as indicated by the decreased number of BrdU positive cells in chronically stressed brain compared to the non-stressed controls.

### Proteome Profile for Anxiety and Related Disorders in Zebrafish Brain Implicates Mitochondrial Dysfunction

Based on 2DE analysis, a total of 18 proteins were identified as differentially regulated in the zebrafish brain tissue in response to CUS based on the observation of 1.5-fold changes (Table 4). The identified proteins are associated with small molecule metabolism, phosphocreatine metabolism and nucleotide metabolism. Most of the brain proteins altered in zebrafish anxiety and related disorders are regulators of mitochondrial function, glycolysis and oxidative stress. Proteins such as ALDOAA, PGAM1B, PHB2, SLC25A5, SNCB, TPI1B, VDAC3, alpha globin, PPIAB and IDH2 were found to be upregulated, whereas ALDOCB, CKM, EEF1A, CH211236K19.6, NME2B, PVALB5 and CKB were found to be downregulated relative to their levels in control brain samples (Table 4). We found that some proteins, such as aldolase C, synuclein B and PVALB2, which have been implicated in mood disorders in rodents [34]–[36] and in other psychiatric disorders in humans [37]–[39], were also dysregulated in zebrafish. The interesting protein ependymin (EP), a fish neurotrophin involved in neuroplasticity, was also observed in our proteome data; it was down-regulated (1.5-fold) in the brains of fish exhibiting CUS-induced anxiety and related disorders. Another protein that was dysregulated in zebrafish brain was SLC25A5 (solute carrier family 25 member 5), which has been linked to autism [40] and Parkinson’s disease [41]. Similarly, eEF1A (elongation factor 1 alpha), which is a translation regulator implicated in neurodegeneration associated with muscle wastage [42], was also found to be dysregulated in stressed fish brain in our study.

**Table 4:** List of proteins identified as differentially regulated between control and stress zebrafish brain tissue based on 2DE MS/MS analysis.

Accession	Gel spot ID	Control	Stressed	Description	Symbol	Score	Coverage	# Peptides	# PSMs	MW [kDa]	calc. pI
IPI00509500.2	18	1	2.7	Fructose-bisphosphate aldolase	ALDOAA	80.47	43.96	10	23	39.7	8.19
IPI00490850.1	15	1	0.3	Fructose-bisphosphate aldolase C-B	ALDOCB	54.57	32.51	6	14	39.2	6.64
IPI00482319.2	14	1	0.3	Muscle-specific creatine kinase	CKM	45.86	17.85	5	12	42.8	6.8
IPI00512240.1	44	1	0.5	Elongation factor 1-alpha	eEF1A	60.86	27.06	7	16	50	9.09
IPI00611053.1	24	1	1.8	Phosphoglycerate mutase	PGAM1B	56.13	34.25	7	14	28.8	6.65
IPI00501947.1	22	1	2.5	Prohibitin 2	PHB2	20.29	17.22	4	6	33.3	9.91
IPI00492110.2	22	1	2.5	Solute carrier family 25, member 5	SLC25A5	20.16	18.12	4	6	32.7	9.76
IPI00482052.1	39	1	1.5	Beta-Synuclein	SNCB	56.08	21.26	2	12	13.3	4.45
IPI00919385.1	38	1	1.7	Triosephosphate isomerase B [Danio rerio]	TPI1B	26.42	37.88	5	7	21.8	5.91
IPI00851926.2	21	1	1.5	Voltage-dependent anion channel 3	VDAC3	90.38	33.12	6	25	32.8	8.91
IPI00486019.2	36	1	0.3	Peptidyl-prolyl cis-trans isomerase	CH211-236K19.6	10.64	20.5	3	3	17.9	7.08
IPI00490492.2	43	1	0.4	Parvalbumin isoform 2a	PVALB5	7.99	19.44	2	2	11.8	4.86
IPI00933179.1	42	1	0.4	Nucleoside diphosphate kinase B	NME2B	7.05	23.21	2	2	12.6	5.2
IPI00497388.2	5	1	0.4	Creatine kinase, brain b	CKB	421.56	43.57	14	133	42.9	5.8
IPI00491770.1	32	1	1.5	Alpha globin like	HBAA1	32.45	49.65	6	9	15.6	8.85
IPI00497774.1	35	1	1.8	Peptidyl-prolyl cis-trans isomerase	PPIAB	65.26	54.88	6	19	17.5	8.07
IPI00500416.1	16	1	2.3	Isocitrate dehydrogenase 2 (NADP+), mitochondrial	IDH2	204.87	33.18	11	63	50.4	8.12
IPI00481812.2	30	1.0	1.5	Ependymin	EPD	76.55	22.12	3	20	24.5	5.64

This table provides the list of 18 proteins identified as differentially regulated between the control and stressed zebrafish brains. The proteins are listed along with their accession numbers, gel spot IDs, fold change in control, fold changes in stressed fish, descriptions, protein symbols, scores, coverages, numbers of peptides identified, peptide sequence matches (PSM), molecular weights, and calculated pI.

**Figure 3:**
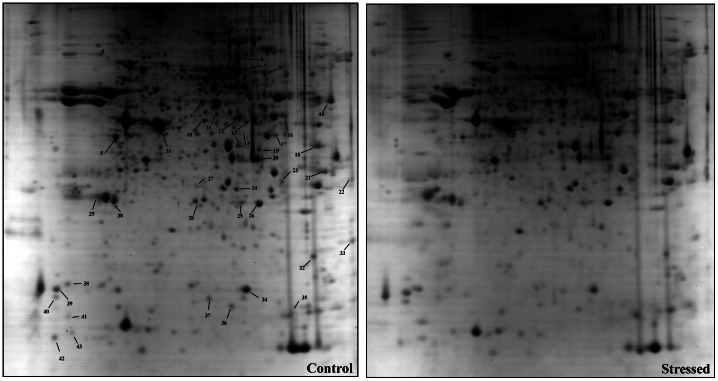
Two-dimensional gel electrophoresis of zebrafish brain shows differential protein levels in response to CUS. Two-dimensional gel electrophoresis pattern of zebrafish brain (a, control and b, stress group) visualized with fast Coomassie staining. Proteins were separated according to the pI gradient (x-axis) in the first dimension and the molecular weight in the second dimension (y-axis). Eighteen spots (labeled in control gel) were identified as differentially regulated under CUS and analyzed by tandem mass spectrometry (MS/MS).

**Figure 4:**
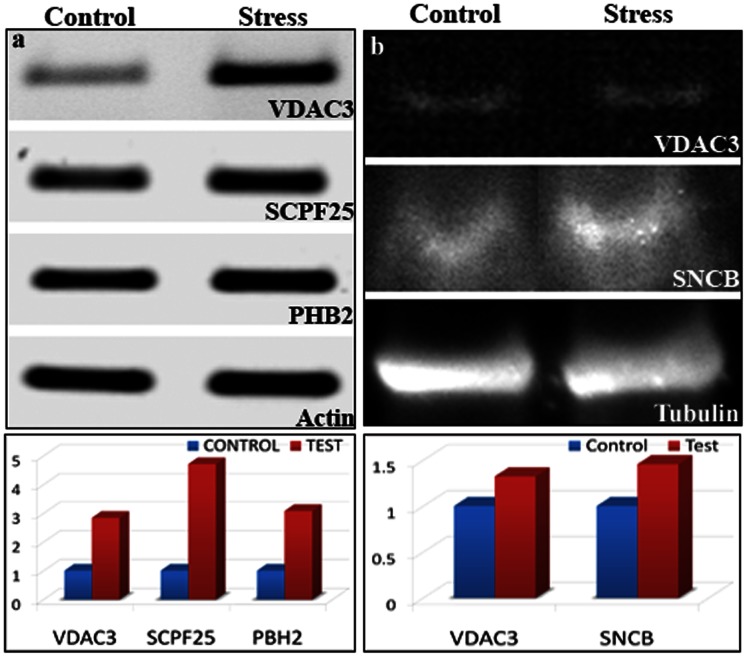
Validation of proteomic results through immunoblotting and gene expressions for a subset of corresponding proteins. CUS upregulates VDAC3, PHB2 and SLC25A5 gene expression levels, as indicated by the fold changes at the mRNA level (a). Similarly, we validated the stress regulation of VDAC3 & SNCB (2 identified proteins from the proteomic list), as observed in the immunoblot result (b).

Two of the 18 proteins identified in our study–voltage-dependent anion channel 3 (VDAC3) and beta-synuclein (SNCB)–were validated using immunoblotting (Figure 3a). Immunoblotting was also performed to assess whether pCREB, a central brain molecular marker in the rodent mood disorder model, is also relevant in the zebrafish model. As in mouse and rat brain, the pCREB level in zebrafish brain was remarkably reduced following CUS (Figure 3b). Quantitative PCR was also performed to validate prohibitin 2 and SLC25A5, two of the 18 proteins found to be maximally regulated (increased by 2.5-fold) in our proteomic data; these proteins regulate mitochondrial function and are implicated in neuropsychiatric and neurodegenerative disorders [27], [40], [43]. The mRNA levels of the genes coding for prohibitin 2 and SLC25A5 were also significantly increased (2.5-fold) in fish that developed anxiety and related phenotypes following chronic stress exposures (Figure 4).

## Discussion

Chronic stress-induced depression, anxiety and related mood disorder models in rodents have yielded a number of molecular markers for central nervous system (CNS) drug screening to develop better therapeutics. However, CNS drug discovery is quite costly when rodent models are employed. We therefore investigated alternative *in vivo* models of anxiety and related affective disorders that mimic the phenotypes of rodent and human mood disorders. Moreover, zebrafish are easy to handle and very economical for screening compound libraries for CNS drug discovery. In this context, we aimed to develop a zebrafish model for anxiety and related affective disorders. The CUS paradigm appears to be very robust in inducing an anxiety and related mood disorder phenotype in zebrafish. Until now, few laboratories [15] have reported the induction of an anxiety phenotype in zebrafish [18] using chronic stress paradigms. Here, we followed the same CUS paradigm for inducing mood disorder phenotypes. However, unlike Piato *et al*. [15], the behavioral assays in this investigation were extensive and allowed the extent of mood disorders in zebrafish induced by repeated stressful events to be characterized (Figure 1a - 1j). The behavioral analyses revealed that the CUS paradigm successfully caused severe anxiety in fish, as shown by the disruption in the normal swimming zone preference [17], increased duration in shoal cohesion and decreased latency for shoal formation, increased duration in dark chamber and reduced latency to enter the dark chamber; all of these behaviors reflect an anxiety phenotype [44]. In addition, the fish exhibited significant numbers of freezing bouts, which can be a PTSD-like phenotype, similar to what researchers have shown with regard to the panic (freezing) response following exposure to olfactory cues (alarm substances) emanating from conspecifics’ macerated tissues and blood [12], [22], [23]; however, in our study, we did not use any particular cue. Moreover, in support of our finding of increased freezing behavior, many researchers have shown similar phenotypes in zebrafish, i.e., reduced exploration or longer latency to reach the top level of the tank, fewer entries to the top, longer and more frequent freezing behaviors and elevated levels of erratic movement [12], [45]–[47]. Further investigation of the PTSD phenotype in zebrafish upon re-exposure to cues or context will be interesting.

Once it became evident that the 15-day CUS paradigm was successful at inducing an anxiety and related disorder phenotype, we aimed to determine whether some of the well-established brain molecular markers of the rodent mood disorder model were applicable to the zebrafish model of CUS-induced anxiety and related mood disorders. Interestingly, CRF and calcineurin showed changes, i.e., significant upregulation in zebrafish brain (Figure 2a), similar to those observed in the brain of rodent models of mood disorders [3], [29]. However, one very well-established molecular marker in depressed rodent brain, BDNF, showed no significant down-regulation in our zebrafish study, although such down-regulation is commonly observed in rodent models [2], [30]. This finding may have been observed because we used whole fish brains for gene expression analysis rather than specific brain areas. Recent studies on rodents have shown varying levels of BDNF in different brain regions that persist for different durations after different stress paradigms [48]–[50]. Therefore, future investigations using specific regions of the zebrafish brain might generate a clear picture of whether the BDNF level also varies in response to stress in a region-specific manner in zebrafish brain. Our validation of the relevance of the molecular stress response markers seen in rodent models, such as CRF and calcineurin, in zebrafish supports the stress hypothesis that was previously developed in rodents [51], [52]. Another well-established molecular marker, pCREB, which was shown to be downregulated in human fibroblasts from patients with major depression and in the postmortem brains of suicide victims with a history of depression [53], was also found to be significantly attenuated in zebrafish brain in CUS-induced affective disorders in our study (Figure 2b).

We also investigated the effects of CUS on adult neurogenesis in the zebrafish telencephalon and optic tectum, the two most neurogenic niches in teleost fish brain, as reduced neurogenesis is a hallmark of anxiety and related mood disorders in rodent models. Our results showed that neurogenesis was severely affected by CUS in a highly neurogenic area of fish brain, the optic tectum (Figure 2c, 2d & 2e), as cells positive for the cell proliferation marker BrdU [54] were drastically reduced in repeatedly stressed brain samples compared to samples from non-stressed control brains. There was also attenuation in the proliferative populations of the telencephalon (Figure S1a, b). Reduced neurogenesis affects hippocampal neuroplasticity in rodent models and leads to anxiety, depression and related mood disorders, as well as cognitive disorders [38], [55]. It will be interesting to investigate the implications of this reduction in neural progenitor proliferation in different brain areas in the zebrafish model.

Once we established the anxiety and related mood disorders model in zebrafish, we sought to uncover molecular markers to facilitate understanding the etiopathology at the molecular level and the screening of a compound library generated by chemists at our institute. We used a simple 2DE proteomics approach to identify 18 differentially regulated proteins in zebrafish brain (Table 3). Interestingly, most of these proteins are involved in mitochondrial function, glycolysis, oxidative stress and the hypoxia stress response. However, we also identified a few proteins, such as aldolase C and beta synuclein (SYNB), that have recently been implicated in depression, anxiety and other affective disorders in rodent models [34].

The proteins aldolase C, beta synuclein and parvalbumin isoform 2 (PVALB2) are implicated in neurological conditions such as Alzheimer’s, Parkinson’s [56], Ischemia [57] and autism [58], as well as in mood disorders [34]–[37]. Furthermore, one interesting protein identified in our study, the fish neurotrophic factor ependymin (EPD), was upregulated in the CUS group. This protein has been found to have higher expression levels in sub-dominant rainbow trout than in dominant trout, and lower expression levels of this protein are correlated with aggression in these fish [59]. EPD might be involved in stress response, as sub-dominants experience stress in the social order. Similarly, another interesting protein that was significantly downregulated in the CUS response was the translation regulator eEF1A, which was also found to be differentially regulated in zebrafish brain in response to microcystin toxicity [60]. Inhibiting eEF1a rendered the cardiomyocyte cell line H9C2 resistant to H_2_O_2_ and ER-stress-induced cell death via actin cytoskeleton remodeling [61]. The reduced eEF1A protein level in zebrafish brain following CUS suggests that this protein plays a role in neuroprotection. Interestingly, this protein is also involved in the neurodegeneration associated with muscle wasting in mice [42]. Another major category of proteins that was affected in our study comprises the proteins involved in glycolysis and brain metabolism: ALDOAA, ALDOCB, PGAM1B, TPI1B, CKM and CKB. Because fish live in water and their swimming patterns are drastically altered in response to stress, the metabolic energy requirements change, which may be the reason for the altered levels of these proteins. Another interesting protein identified in our study, PPIAB (peptidyl-prolyl cis-trans isomerase), is involved in multiple functions, such as protein folding, signal transduction, trafficking, assembly and cell cycle regulation; these processes are critical for the neuroglial and behavioral adaptations that always underlie anxiety and related mood disorders [34].

However, the most remarkable finding of our study was the mitochondrial dysfunction that occurred in response to CUS. It is noteworthy that mitochondrial functions are compromised in many neurological and psychiatric disorders [62]. This study identified 4 out of 18 total proteins that are involved in mitochondrial function, PHB2, SLC25A5, VDAC3 and IDH2, as differentially regulated. PHB2 (prohibitin 2) is implicated in aging. It is a mediator of transcriptional repression by nuclear hormone receptors, particularly estrogen receptor (ER) and is found associated with histone deacetylase (HDAC) and nuclear receptor co-repressor 1 (NCoR1). SLC25A5, a solute carrier family 25 alpha member 5 protein, also known as ANT2, is a member of the mitochondrial carrier subfamily of solute carrier protein genes and functions as a gated pore, translocating ADP from the mitochondrial matrix into the cytoplasm to maintain the cytoplasmic phosphorylation potential for cell growth. Suppressing the expression of this gene has been shown to induce apoptosis and inhibit tumor growth. Here, while validating the proteomics data, we found that the protein level of SLC25A5 increases substantially (4.0-fold increase in the protein level; 3.5-fold increase in the mRNA level) in stressed fish brain. This finding suggests the existence of a compensatory mechanism to protect the cells from stress-induced damage. IDH2 (isocitrate dehydrogenase) is an NADP(+)-dependent mitochondrial protein that regulates mitochondrial redox status through its role in intermediary metabolism and energy production. IDH2 appears to be tightly associated with the pyruvate dehydrogenase complex. Recently, Sirt3, one of three mitochondrial sirtuins, has been shown to deacetylate lysine 413 on IDH2, stimulating its activity and protecting the cell from oxidative stress [63]. In our study, the 2.3-fold increases in the protein level of IDH2 in stressed fish brain might represent a protective strategy against oxidative stress. VDAC3 (voltage-dependent anion channel 3) belongs to the mitochondrial porin family and allows ATP and other small metabolites to cross the membrane. This protein appears to form part of the mitochondrial permeability transition pore for the release of cytochrome c and the induction of apoptotic cell death [27]. The increased level of VDAC3 in stressed fish brain might increase the efficiency of bioenergetic metabolism and/or protection against reactive oxygen species (ROS) [64]. In summary, alteration of the brain proteome profile in response to CUS indicates that mitochondrial dysfunction underlies the anxiety and related mood disorder phenotype in zebrafish.

The zebrafish CUS model can enable CNS drug discovery in two ways: First, this simple stress-induced anxiety and related affective disorder model can be used for the routine screening of compounds positive in cell-based assays for CNS drug discovery, as well as for *in vivo* validation of the lead molecules for their efficacy in various neurological and psychiatric disorders. Second, a better understanding of stress-induced alterations in the brain proteome profile might facilitate the identification of new molecular targets for CNS drug development.

## Supporting Information

Figure S1 **CUS affects neurogenesis negatively in adult zebrafish.** Neurogenesis in the telencephalon, which corresponds to the hippocampal neurogenic region in rodents, is moderately affected after CUS; the number of BrdU positive cells is reduced in stressed brain sections (b) compared to the non-stressed control (a).
(TIF)
