# Supplementary figures and images for: Chronic Unpredictable Stress (CUS)-Induced Anxiety and Related Mood Disorders in a Zebrafish Model: Altered Brain Proteome Profile Implicates Mitochondrial Dysfunction
Source: PLoS One. 2013 May 14;8(5):e63302. doi: 10.1371/journal.pone.0063302 (PMC3653931; doi:10.1371/journal.pone.0063302)

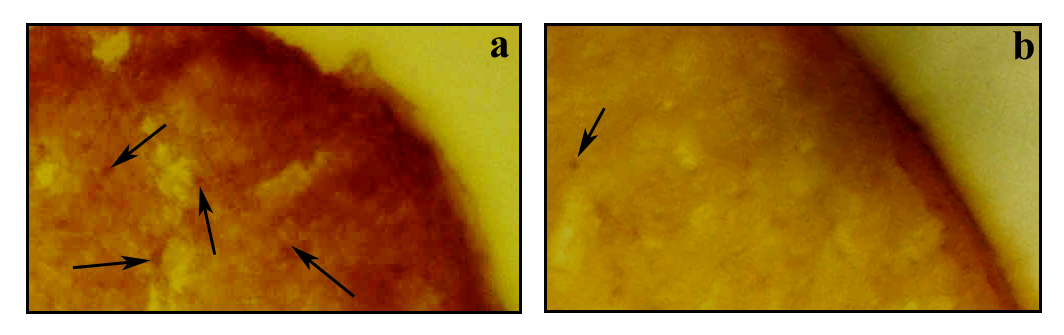

Supplement: Figure S1 — CUS affects neurogenesis negatively in adult zebrafish. Neurogenesis in the telencephalon, which corresponds to the hippocampal neurogenic region in rodents, is moderately affected after CUS; the number of BrdU positive cells is reduced in stressed brain sections (b) compared to the non-stressed control (a). (TIF) [file pone.0063302.s001.tif]
